# Promoter trap strategy for gene expression analysis under stress conditions of *M. tuberculosis* latency

**DOI:** 10.1186/1471-2334-14-S3-O13

**Published:** 2014-05-27

**Authors:** Anant Yadav, Shivani Sood, Rahul Shrivastava

**Affiliations:** 1 Department of Biotechnology and Bioinformatics, Jaypee University of Information Technology, Waknaghat, Solan-173234, Himachal Pradesh, India

## Background

Tuberculosis, caused by *Mycobacterium tuberculosis*, still remains a significant bacterial killer globally accounting for ~ 2 million deaths annually. The uniqueness of the infection lies in the competence of the etiological agent to remain dormant in the human body for extended periods of time, escaping the body’s defense mechanisms. The ineffectiveness of the currently used antimycobacterials against latent mycobacteria warrants for discovery of novel drugs and drug targets. New methods are also required for the identification, analysis and validation of drug targets against latent mycobacteria.

## Methods

A promoter trap vector with *lacZ* reporter system was constructed. For validation of the vector, upstream sequences of genes known to be involved in *M. tuberculosis* latency along with suitable positive and negative controls were PCR amplified and cloned into the shuttle vector containing promoterless *lacZ* reporter system, to yield recombinant constructs. The recombinant constructs were then electroporated into *M. smegmatis* to obtain recombinant mycobacterial strains. All such strains were individually subjected to stress conditions associated with *M. tuberculosis* latency.

## Results

Validation of the promoter trap vector was done by expression analysis of *lacZ* reporter using a β-galactosidase assay. Genes known to be involved in the latency of *M. tuberculosis* showed appreciable levels of β-galactosidase expression under anaerobiosis as well nutrient starvation conditions in comparison to the negative control strain.

## Conclusion

This promoter trap strategy can be exploited for expression analysis and validation of drug targets deduced from various non conclusive strategies such as bioinformatic analysis and microarray. This can also be utilized for determination of new drug targets by construction of genomic library.

